# Integrated Multi‐Omics Analysis of *Polygonatum odoratum* Polysaccharide in D‐Galactose‐Treated Mice Reveals Changes in Oxidative Stress Markers and Gut Microbiota

**DOI:** 10.1002/fsn3.72500

**Published:** 2026-10-28

**Authors:** Wenjian Li, Yan Huang, Wenfang Zhang, Shunxiang Li, Lisong Li

**Affiliations:** ^1^ School of Pharmacy Hunan University of Chinese Medicine Changsha China; ^2^ Hunan Province Engineering Technology Research Center of Bioactive Substance Discovery of Chinese Medicine Changsha China; ^3^ Hunan Province Hospital of Integrated Traditional Chinese and Western Medicine (Affiliated Hospital of Hunan Academy of Chinese Medicine) Changsha China; ^4^ Hunan Province Sino‐US International Joint Research Center for Therapeutic Drugs of Senile Degenerative Diseases Changsha China; ^5^ School of Informatics Hunan University of Chinese Medicine Changsha China

**Keywords:** aging, D‐galactose, multi‐omics analysis, oxidative stress, *Polygonatum odoratum* polysaccharide

## Abstract

Age‐related changes are associated with oxidative stress, metabolic dysregulation, and alterations in the gut microbiota. Although *Polygonatum odoratum* polysaccharide (POP) has shown antioxidant and neuroprotective effects, its associations with gut bacterial composition, circulating metabolites, and tissue gene expression remain unclear. Using a D‐galactose‐treated mouse model, we assessed hippocampal histology and oxidative stress markers and performed bacterial 16S rRNA sequencing, untargeted serum metabolomics, and hippocampal and colonic transcriptomic analyses. POP treatment was associated with improved hippocampal histology, reduced malondialdehyde levels, and increased glutathione levels and superoxide dismutase activity. It was also associated with changes in selected gut bacterial taxa and serum lipid metabolites, lower expression of NF‐κB‐related genes (*Btk*, *Lat*, and *Tnfrsf13c*) in the colon, and lower *Ninj2* expression in the hippocampus. Cross‐omics analysis identified correlations among these bacterial, metabolite, and gene‐expression features. These findings identify candidate gut–serum–tissue associations accompanying the effects of POP in D‐galactose‐treated mice. Functional studies are needed to determine whether any of these changes mediate its effects.

## Graphical abstract

This study evaluated the effects of *Polygonatum odoratum* polysaccharide (POP) in D‐galactose‐treated mice using hippocampal histology, oxidative stress measurements, and integrated multi‐omics analyses. Results showed improved hippocampal histology and oxidative stress markers, accompanied by changes in gut bacterial composition, serum metabolites, and hippocampal and colonic gene expression. Cross‐omics correlations highlighted candidate gut–serum–tissue associations for further functional investigation.

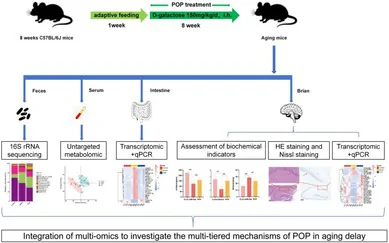

## 1 Introduction

Aging is characterized by progressive physiological decline, disrupted tissue homeostasis, and chronic inflammation and oxidative stress, leading to increased risk of neurodegenerative and metabolic disorders (Hou et al. 2019; Kroemer et al. 2025). With global population aging accelerating, identifying safe and effective anti‐aging interventions has become a major challenge (Dong et al. 2026; Jiang et al. 2025). Oxidative stress, metabolic alterations, and gut microbiota dysbiosis are key drivers of aging (Wang et al. 2025; Yang et al. 2025). However, aging involves coordinated multi‐system changes that cannot be captured by single‐molecule or single‐pathway approaches, limiting our understanding of the holistic regulatory effects of natural products.

Recent advances in integrated multi‐omics technologies (e.g., microbiomics, metabolomics, transcriptomics) have enabled systematic dissection of aging and its intervention mechanisms (Behrens et al. 2026; Ren et al. 2026). This approach allows holistic mapping of interactions among gut microbiota, host metabolites, and tissue gene expression, revealing the role of the gut–organ axis in aging regulation (Yang et al. 2024; Zhao et al. 2025). From a systems biology perspective, the synergistic, multi‐target effects of natural bioactive compounds against aging can thus be more clearly elucidated.

*Polygonatum odoratum* (Yuzhu) is a traditional medicinal and edible plant in China, valued for nourishing Yin, moistening the lungs, and promoting fluid production. Its anti‐aging effects are documented in ancient herbal classics and the modern Chinese Pharmacopeia (Shu et al. 2026). *Polygonatum odoratum* polysaccharide (POP) is its major active component. Previous studies have shown protective effects of *Polygonatum odoratum* polysaccharide (POP) in D‐galactose‐treated animals. In a mouse study, POP was administered at 1 or 2 g/kg/day for 8 weeks, and its effects on learning and memory and brain oxidative stress were evaluated (Peng et al. 2020). In a separate study using D‐galactose‐treated rats, Xiao et al. (2024) administered POP at 200, 400, or 800 mg/kg/day for 12 weeks. They assessed cognitive performance using the Morris water maze and open‐field test and examined hippocampal pathology, ferroptosis‐related markers, and Nrf2 signaling. These studies established a basis for investigating POP in experimental aging models. The present study extends this work by examining whether POP treatment is accompanied by changes in gut bacterial composition, circulating metabolites, and hippocampal and colonic gene expression within the same experimental framework.

We hypothesized that, in D‐galactose‐treated mice, POP‐associated improvements in hippocampal histology and oxidative stress markers would coincide with changes in gut bacterial composition, serum metabolites, and hippocampal and colonic gene expression. We further examined whether selected bacterial taxa, circulating metabolites, and tissue transcripts showed cross‐omics correlations, with particular attention to colonic NF‐κB‐related genes and hippocampal *Ninj2*. To address these questions, we combined bacterial 16S rRNA sequencing, untargeted serum metabolomics, and hippocampal and colonic transcriptomics. This design identifies candidate associations for subsequent functional testing.

## 2 Materials and Methods

### 2.1 Preparation of POP

The YuZhu used in this study were obtained from the National Good Agricultural Practice (GAP) Base for YuZhu in Shaodong County, Hunan Province, China. All experimental samples were authenticated as *Polygonatum odoratum* (Mill.) Druce (family Liliaceae) by Professor Liu Tasi from the Department of Identification of Chinese Materia Medica, Hunan University of Chinese Medicine. The extraction of POPs is based on our previous research (Xiao et al. 2024). POP was obtained from Yuzhu via water extraction, ethanol precipitation, and deproteinization, with a yield of 5%. The total sugar content of POP, determined by the phenol‐sulfuric acid method, was 75.08% ± 0.55%. The main POP components were analyzed using high‐performance liquid chromatography (HPLC; 1260 Infinity, Agilent, Santa Clara, CA, USA). The mobile phase consisted of acetonitrile and 0.2% ammonium acetate (23:77, v/v). The flow rate was 1.0 mL/min, the column temperature was 30°C, the injection volume was 10 μL, and the detection wavelength was 250 nm. Liquid chromatography analysis revealed that POP was composed of mannose, rhamnose, glucose, galactose, and arabinose, with relative molar percentages of 12.76%, 1.17%, 77.74%, 3.38%, and 4.95%, respectively (Figure 1).

**FIGURE 1:**
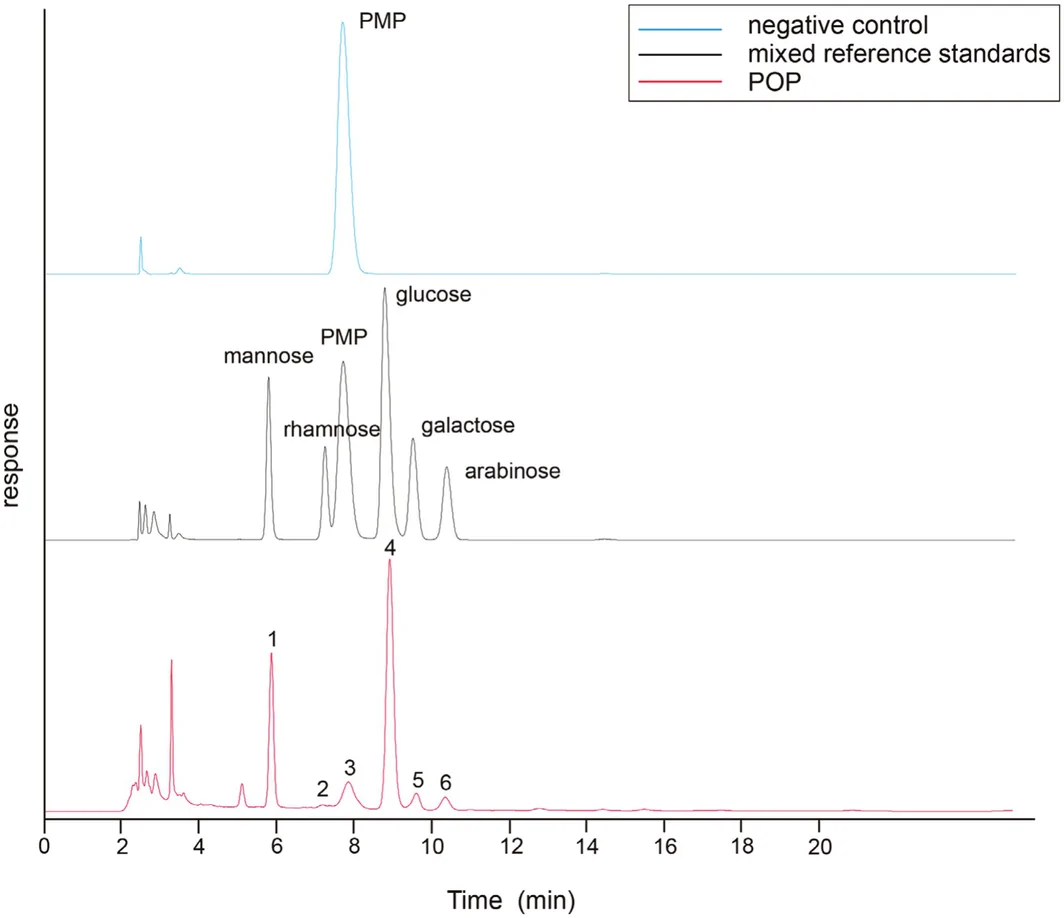
Monosaccharide composition of POP. POP was composed of mannose, rhamnose, glucose, galactose, and arabinose, with relative molar percentages of 12.76%, 1.17%, 77.74%, 3.38%, and 4.95%, respectively.

### 2.2 Animals and Experimental Treatments

Eight‐week‐old male C57BL/6 mice were purchased from Hunan SJA Laboratory Animal Co. Ltd. (Hunan, China). The mice were housed in a controlled environment at 22°C, with 55% relative humidity and a 1:1 light/dark cycle. Food and water were provided ad libitum.

The mice were randomly assigned to three groups (*n* = 10): Control, Model, POP. Each group was housed in two cages, with five mice per cage. Cages were randomly assigned to the Control, Model, and POP groups, and animals from different experimental groups were housed in separate cages to avoid cross‐contamination. All cages were maintained in the same animal room under identical environmental conditions. Mice in the POP and Model groups were subcutaneously injected with D‐galactose (150 mg/kg) daily, while the Control group received an equivalent volume of saline. Meanwhile, the POP group was administered POP at 4 g/kg body weight per day via oral gavage, and the Control group received an equivalent volume of saline. The POP group received POP at 4 g/kg body weight per day by oral gavage for 8 weeks. This dose was selected based on our preliminary observations in D‐galactose‐treated mice. The present study used a single POP dose to examine histological, biochemical, and multi‐omics outcomes within the same experimental framework. All treatments were continued for 8 weeks.

After 8 weeks of treatment, the mice were fasted overnight and sacrificed. Fecal, serum, hippocampal, and colonic samples were collected and stored at −80°C. Fecal and serum samples from each group were used for 16S rRNA sequencing (*n* = 6) and untargeted metabolomic (*n* = 5) detection, respectively. Hippocampal and colonic samples from each group were used for transcriptomic (*n* = 3) and quantitative real‐time polymerase chain reaction (PCR) analysis (*n* = 3).

### 2.3 Fecal 16S rRNA Sequencing and Processing

Each mouse was kept in a sterile cage for 24 h to collect fecal samples. For fecal collection only, each mouse was temporarily housed individually in a sterile cage for 24 h; routine housing during the treatment period was as described in Section 2.2. A total of 18 samples were collected and subjected to 16S rRNA gene amplicon sequencing (Bioyi Biotechnology Co. Ltd., Wuhan, China). Total genomic DNA was extracted using the OMEGA Soil DNA Kit (M5635‐02, Omega Bio‐Tek, Norcross, GA, USA) following the manufacturer's instructions, and DNA extracts were stored at −20°C. DNA quantity and quality were assessed using a NanoDrop NC2000 spectrophotometer (Thermo Fisher Scientific, Waltham, MA, USA) and agarose gel electrophoresis. The V3–V4 region of the bacterial 16S rRNA gene was amplified using primers 338F (5′‐ACTCCTACGGGAGGCAGCA‐3′) and 806R (5′‐GGACTACHVGGGTWTCTAAT‐3′), with sample‐specific 7‐bp barcodes for multiplexing. The PCR mixture (25 μL) contained 5 μL of 5× buffer, 0.25 μL of Fast pfu DNA Polymerase (5 U/μL), 2 μL of dNTPs (2.5 mM), 1 μL of each primer (10 μM), 1 μL of DNA template, and 14.75 μL of ddH_2_O. Thermal cycling included initial denaturation at 98°C for 5 min, followed by 25 cycles of 98°C for 30 s, 53°C for 30 s, and 72°C for 45 s, with a final extension at 72°C for 5 min. PCR amplicons were purified using Vazyme VAHTSTM DNA Clean Beads (Vazyme, Nanjing, China) and quantified with the Quant‐iT PicoGreen dsDNA Assay Kit (Invitrogen, Carlsbad, CA, USA). Equimolar amounts of amplicons were pooled and subjected to paired‐end sequencing (2 × 250 bp) on the NovaSeq 6000 platform (Illumina, San Diego, CA, USA) at Bioyi Biotechnology Co. Ltd. (Wuhan, China). Raw paired‐end reads were processed using QIIME2. Adapters and primers were removed with the cutadapt trim‐paired plugin, followed by quality filtering, denoising, read merging, and chimera removal using the dada2 denoise‐paired plugin with default parameters.

### 2.4 Untargeted Metabolomic Analysis of Serum

Serum samples (100 μL) were mixed with prechilled 80% methanol, incubated on ice for 5 min, and centrifuged at 15,000*g*, 4°C for 20 min. The supernatant was diluted with LC–MS grade water to 53% methanol, then centrifuged again under the same conditions. The final supernatant was analyzed by UHPLC–MS/MS.

UHPLC–MS/MS was performed on a Vanquish UHPLC system coupled to an Orbitrap Exploris 120 mass spectrometer (Thermo Fisher) in Bioyi Biotechnology Co. Ltd. Wuhan, China. Separation used a Hypersil Gold column (100 × 2.1 mm, 1.9 μm) at 0.2 mL/min with a 12‐min linear gradient. Mobile phases: positive mode—A: 0.1% formic acid in water, B: methanol; negative mode—A: 5 mM ammonium acetate (pH 9.0), B: methanol. Gradient: 2% B for 1.5 min, 2%–85% B for 3 min, 85%–100% B for 10 min, then back to 2% B. MS operated in positive/negative polarity with spray voltage 3.5 kV, capillary temperature 320°C, sheath gas 35 psi, auxiliary gas 10 L/min, S‐lens RF 60, auxiliary heater 350°C.

Raw data (.raw files) were processed using Compound Discoverer 3.3 (CD3.3, Thermo Fisher Scientific) for peak alignment, peak picking, and quantification. The main parameters were set as follows: actual mass tolerance, 5 ppm; signal intensity tolerance, 30%; and minimum signal intensity. Peak areas were corrected using the first QC sample. Missing values were handled using the default settings of Compound Discoverer 3.3. No additional imputation was performed beyond the software default. Peak intensities were normalized to the total spectral intensity. Metabolites were identified by matching molecular formulas predicted from adduct ions, molecular ion peaks, and fragment ions against the mzCloud, mzVault, and MassList databases. Blank samples (53% methanol–water, processed identically to experimental samples) were used to remove background ions.

QC samples were prepared by pooling equal volumes from all experimental samples. The relative peak area of each metabolite was calculated as: relative peak area = sample raw quantitation value/(sum of sample metabolite quantitation values/sum of QC1 sample metabolite quantitation values). Compounds with a coefficient of variation (CV) > 30% in relative peak areas across QC samples were removed. Statistical analyses were performed using R (version 3.4.3), Python (version 2.7.6), and CentOS (release 6.6). Annotations used KEGG, HMDB, and LIPID Maps. PCA and PLS‐DA were performed with metaX. Differential metabolites were defined by VIP > 1, *p* < 0.05 (*t*‐test), and fold change ≥ 2 or ≤ 0.5. Volcano plots, heatmaps (z‐score), and Pearson correlation matrices were generated with R packages. Pathway enrichment analysis used KEGG; significance threshold *p* < 0.05.

### 2.5 Transcriptomic Analysis

Total RNA was extracted from the hippocampal and colonic tissues using TRIzol Reagent (Invitrogen Life Technologies). The extracted RNA was then qualified and quantified as follows: (1) RNA purity and concentration were assessed using a NanoDrop spectrophotometer (Thermo Scientific) and a Qubit 4.0; (2) RNA integrity and quantity were measured using the Agilent 2100/4200 system.

Three micrograms of RNA were used as the input material for RNA sample preparation. Sequencing libraries were generated using the following steps: First, mRNA was purified from the total RNA using poly T oligo‐attached magnetic beads. The purified mRNA was then fragmented using divalent cations under elevated temperatures. First‐strand cDNA synthesis was performed using random hexamers, followed by second cDNA strand synthesis with the addition of buffer, dNTPs, RNase H, and DNA polymerase I. The resulting double‐stranded cDNA was repaired, and an adenine (A) was added to the 3′ end. Hieff NGS DNA Selection Beads were used for purification and fragment selection. After purification and fragment selection, the products were amplified and enriched by PCR. Qubit was used for quantitative.

The target region library of the double‐stranded DNA was denatured, cycled, and digested to generate a single‐stranded circular DNA. The single‐stranded circular DNA was then amplified through rolling circle amplification (RCA) to form DNA nanoballs (DNB). Following library construction, quantification was again performed using Qubit fluorometry. Finally, the sequencing library was subjected to paired‐end sequencing on DNBSEQ‐T7 with PE150 model at Bioyi Biotechnology Co. Ltd. (Wuhan, China).

Raw reads were filtered using fastp (v0.21.0) to remove adapters and low‐quality reads (Q20). Clean reads were aligned to the mouse reference genome GRCm38/mm10 using HISAT2 (v2.1.0). Gene‐level read counts were obtained using StringTie (v2.1.5), and expression levels were normalized as FPKM. Differential expression analysis was performed using DESeq2 (v1.30.1). *p*‐values were adjusted for multiple testing using the Benjamini–Hochberg method. Genes with |log_2_FoldChange| > 1 and *p*adj ≤ 0.05 were considered differentially expressed. GO and KEGG enrichment analyses were performed using clusterProfiler (v3.8.1), with padj ≤ 0.05 considered significantly enriched. GSEA was also performed using clusterProfiler (v3.8.1), and pathways with *p*value < 0.05 and *p*.adjust < 0.05 were considered significantly enriched. Differential alternative splicing events were analyzed using rMATS (v4.1.1). SNPs and InDels were detected using samtools and bcftools. Protein–protein interaction networks were analyzed using the STRING database.

### 2.6 RT‐qPCR

Total RNA was isolated from hippocampal and colonic tissues using the Total RNA Extraction Kit (Vazyme, Nanjing, China), following the manufacturer's instructions. The concentration and purity of the extracted RNA were assessed using a microplate spectrophotometer (Epoch). Subsequently, first‐strand cDNA was synthesized from the total RNA template using the NovoScript Plus All‐in‐One first‐Strand cDNA Synthesis Kit (Novoprotein, Suzhou, China). Gene‐specific primers for target mRNAs were designed with the NCBI Primer‐BLAST tool. Quantitative real‐time PCR was performed using the NovoStart SYBR qPCR SuperMix Plus (Novoprotein, Suzhou, China). Transcript levels were normalized to *β‐actin* and calculated using the $2^{-{\mathrm{\Delta \Delta C}}_{q}}$ method. Primer sequences are presented in Table 1.

**TABLE 1:** Primer sequences used for quantitative real‐time PCR.

Gene	Direction	Primer sequence (5′ → 3′)	Primer length (nt)
*β‐Actin*	Forward	CATCCGTAAAGACCTCTATGCCAAC	25
	Reverse	ATGGAGCCACCGATCCACA	19
*Chrdl2*	Forward	TTTGCTGGGACTCGTGATGTT	21
	Reverse	GTGGTTCCAAGTAGGGGTGC	20
*Mybpc3*	Forward	CAGGGAAGAAACCAGTGTCAG	21
	Reverse	GCTGCCAAACCATACTTGTCATT	23
*Ninj2*	Forward	ATGGAGTCAGACCGAGAAACC	21
	Reverse	TGGCATTGGACATAAAGAGCG	21
*Btk*	Forward	AAGAAGCGCCTGTTTCTCTTG	21
	Reverse	GGTACGGGAACCTTTCAATGAT	22
*Card11*	Forward	GGAGGGCCAGCTATGGATGA	20
	Reverse	GGTTGATGTAACGGCTCAGC	20
*Lat*	Forward	ATGGAAGCAGACGCCTTGAG	20
	Reverse	TGCTGTCATAGGAGACTGGCA	21
*Tnfrsf13c*	Forward	TCTGGTGAGAAACTGCGTGTC	21
	Reverse	GTCAGCGCCAGTATCAGTCC	20

### 2.7 Assessment of Biochemical Indicators

After anesthesia, the hippocampal tissues were rapidly removed, rinsed with cold physiological saline, and the hippocampal tissue was collected on ice. The hippocampal tissue was then homogenized in physiological saline at a mass‐to‐volume ratio of 1:9 to prepare a 10% (w/v) tissue homogenate. The homogenate was centrifuged at low temperature, and the resulting supernatant was collected for protein quantification using a BCA protein assay kit (E‐BC‐K318‐M, Elabscience, Wuhan, China). The levels of total superoxide dismutase (SOD, A001‐3, Nanjing Jiancheng, China), malondialdehyde (MDA, A003‐1, Nanjing Jiancheng), and reduced glutathione (GSH, E‐BC‐K030‐M, Elabscience) were determined following the manufacturer's instructions.

### 2.8 HE and Nissl Staining

Brain tissue samples were fixed in 4% formaldehyde for 48 h, followed by dehydration in 30% sucrose solution for 3 h. The tissues were then embedded in paraffin and sectioned using a rotator microtome to obtain serial sections 5 μm in thickness. The sections were dewaxed with xylene and rehydrated with graded concentrations of ethanol. For HE staining, the sections were stained with hematoxylin and eosin, whereas for Nissl staining, toluidine blue was used.

For quantitative histomorphometric analysis, the hippocampal CA1 region was examined. The percentage of damaged neurons was calculated as the number of damaged neurons divided by the total number of neurons × 100%. Damaged neurons were identified by neuronal swelling, nuclear pyknosis, and irregular alignment. The number of Nissl‐positive neurons in the CA1 region was also counted. Nissl‐positive neurons were defined as intact cells with a clear nucleus and abundant Nissl bodies. For each mouse, measurements from multiple non‐adjacent sections were averaged, and the mouse was used as the independent unit for statistical analysis. All quantifications were performed by an investigator blinded to group allocation using ImageJ software.

### 2.9 Statistical Analysis

Analysis was conducted using GraphPad Prism software (version 9.5.0). Continuous data are presented as the mean ± standard deviation (SD). Because the experimental design involved three independent groups and no repeated measurements, comparisons among the Control, Model, and POP groups were performed using one‐way ANOVA. For multiple comparisons, Tukey's post hoc test was used when variances were homogeneous. When variances were unequal, Tamhane's T2 test was used for pairwise comparisons. Statistical significance was set at *p* < 0.05. Correlation analyses were performed using Pearson correlation for normally distributed variables and Spearman rank correlation for non‐normally distributed variables. Multiple testing correction was performed using the Benjamini–Hochberg false discovery rate (FDR) method, and FDR < 0.05 was considered statistically significant.

## 3 Results

### 3.1 POP Ameliorates Neuronal Damage and Attenuates Oxidative Stress in the Brains of D‐Gal‐Induced Aging Mice

The hippocampus is a key brain region responsible for learning, memory, and spatial navigation. Pathological alterations in the CA1 subregion serve as an important marker of overall brain health, extent of neuronal damage, and associated cognitive impairment. As presented in Figure 2A–D, mice in the Model group exhibited disorganized neuronal arrangement, varying degrees of neuronal damage, and a reduction in cell number. Additionally, neuronal swelling, nuclear pyknosis, a significant decrease in the number of Nissl bodies and nuclei, irregular alignment, and pale staining were observed. In contrast, the mice in the POP group showed improvements compared to those in the Model group, with neuronal recovery, a more orderly arrangement, increased numbers of Nissl bodies and nuclei, relatively regular structure, and deeper, clearer staining. These findings suggest that POP alleviates D‐galactose‐induced neuronal damage in the brain.

**FIGURE 2:**
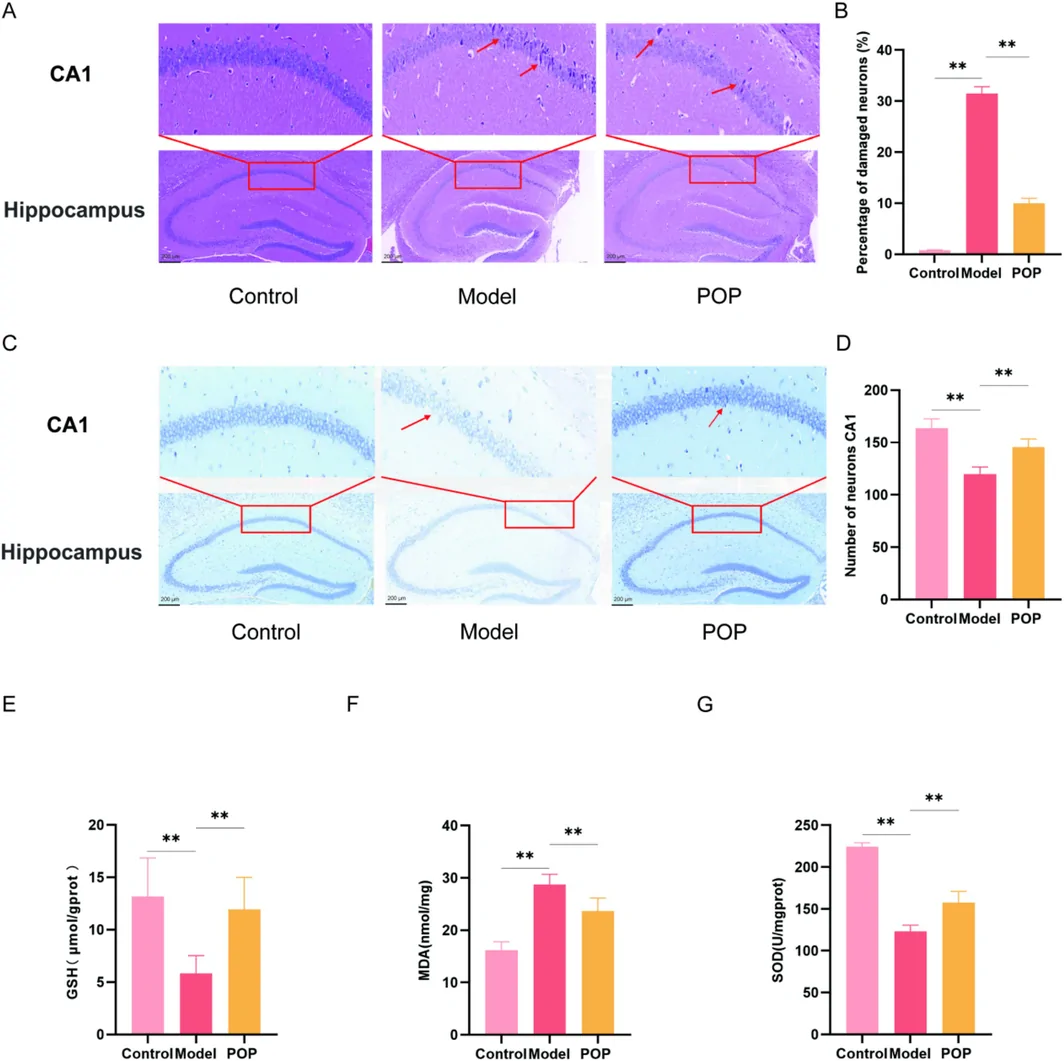
POP ameliorates neuronal damage and alleviates cerebral oxidative stress in D‐galactose‐induced aging mice. (A) Hematoxylin and eosin (H&E) staining of the hippocampal region. (B) Percentage of damaged neurons in CA1 area. (C) Nissl staining of the hippocampal region. (D) Number of positive neurons in CA1 area. (E) Glutathione (GSH) levels. (F) Malondialdehyde (MDA) levels. (G) Superoxide dismutase (SOD) activity. Data are presented as the mean ± SD. *n = 3*, **p < 0.01.

GSH, MDA, and SOD are key indicators for assessing aging‐related damage; therefore, we measured their levels in the brain tissues of mice from each group. As presented in Figure 2E–G, mice in the Model group exhibited significantly increased MDA levels, along with markedly reduced GSH levels and SOD activity in the brain. Conversely, mice in the POP group demonstrated the opposite pattern. These findings indicate that POP mitigates D‐galactose‐induced cerebral oxidative stress.

### 3.2 POP Affects the Abundance of Gut Microbiota in D‐Gal‐Induced Aging Mice

First, we investigated the effect of POP on the gut microbiota using 16S rRNA sequencing. Alpha diversity analysis demonstrated that, while there was no significant difference in species richness among groups based on the Chao1 index, both the Shannon and Simpson indices revealed significant intergroup differences in alpha diversity. These findings suggest that the relative abundance distribution of species is the primary determinant of the observed variations in microbial diversity among groups (Figure 3A–C). NMDS analysis using Bray–Curtis distance metrics indicated notable differences in beta diversity across groups (Figure 3D). At the phylum level, the community abundance analysis demonstrated that Bacteroidota and Firmicutes were the dominant phyla in all three groups of mice (Figure 3E). A heatmap illustrated the relative abundance of various microbes at the genus level, showing that POP treatment ameliorated the aberrant increases or decreases in bacterial taxa observed in aged mice (Figure 3F). Further statistical analysis revealed significantly elevated relative abundances of *Adlercreutzia*_404257, *Allobaculum*, and *Duncaniella* in aged mice, which were markedly reduced by POP treatment (Figure 3G–I). Notably, previous studies have associated *Adlercreutzia* with Alzheimer's disease (Cammann et al. 2023), *Allobaculum* with systemic non‐resolving inflammation under high‐fat diet in aging mice (Kain et al. 2019), and *Duncaniella* with susceptibility to inflammatory bowel disease (Forster et al. 2022). These findings suggest that the abnormal increase of these genera may contribute to an inflammatory and pro‐aging gut microenvironment. POP treatment reversed these changes, indicating a potential modulatory effect on gut microbiota dysbiosis.

**FIGURE 3:**
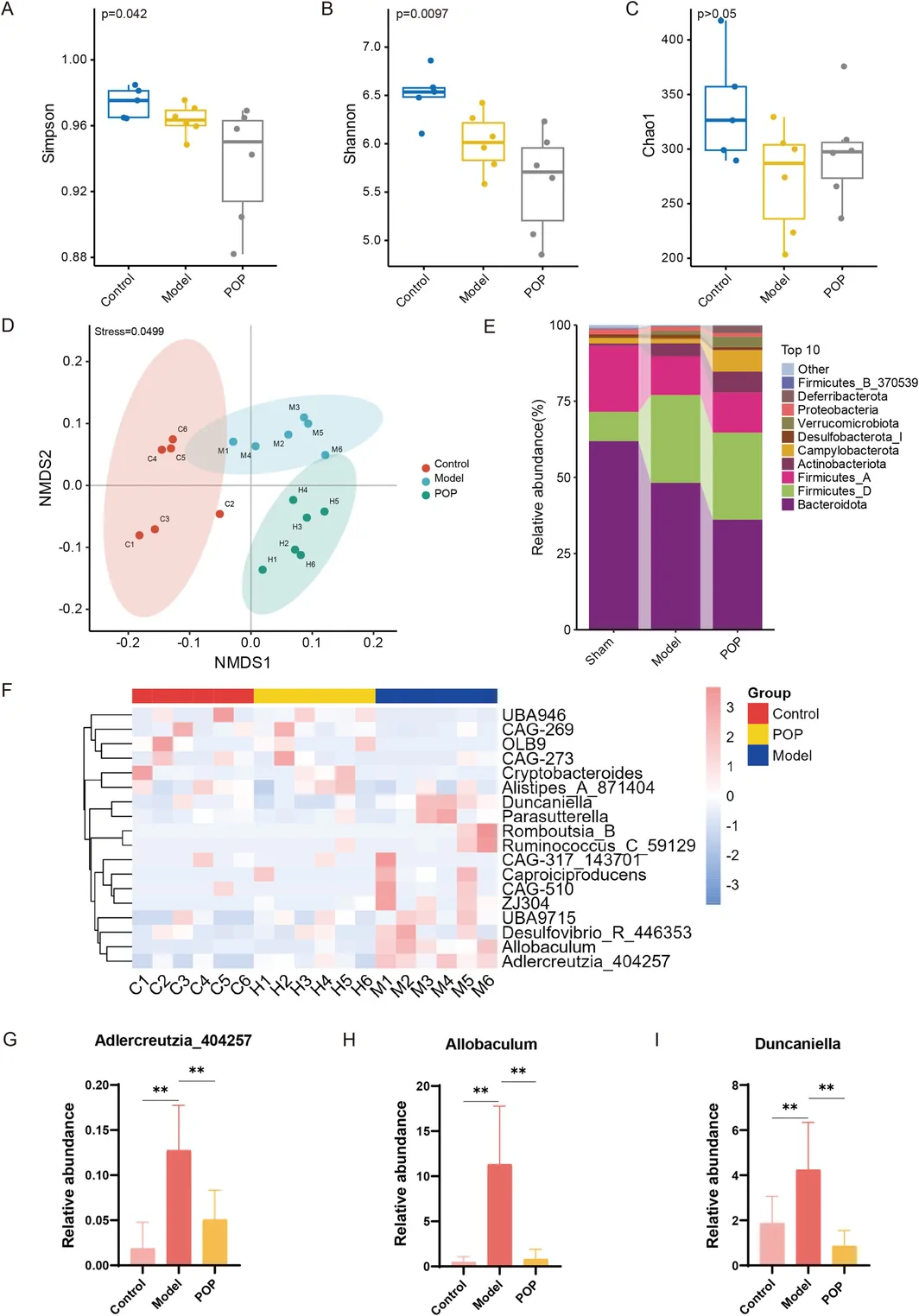
POP affect the abundance of the gut microbiota in D‐galactose‐induced aging mice. (A–C) α‐diversity (Simpson, Shannon and Chao1 indices) of the gut microbiota. (D) Non‐metric multidimensional scaling (NMDS) analysis. (E) Relative abundance of the top 10 bacterial phylum level. (F) Heatmap of microbial abundance at the genus level. (G–I) Three genera showing statistically significant differences in abundance. Data are presented as mean ± SD. *n* = 6. **p < 0.01.

### 3.3 POP‐Mediated Alterations in Serum Metabolites of D‐Gal‐Induced Senescent Mice

The activity of gut microbiota and its resulting effects are directly reflected in the types and concentrations of small‐molecule substances in the blood. Consequently, profiling of serum metabolites can provide valuable insights into gut microbial functions and their systemic impacts on the host. To investigate the effect of POP on metabolism in aging mice, we employed untargeted metabolomics to analyze the changes in serum metabolites across experimental groups. PCA revealed clear separation among the serum metabolomic profiles of each group (Figure 4A). Using the criteria of VIP > 1 and *p* < 0.05, we identified 63 significantly differential metabolites between the Control and Model group, and 41 differential metabolites between the Model and POP group (Figure 4B,C). Pathway enrichment analysis of differential metabolites between the Control and Model groups indicated that these metabolites were primarily involved in glycerolipid metabolism, inositol phosphate metabolism, and secondary metabolite biosynthesis. In contrast, the differential metabolites between the Model and POP groups were significantly enriched in glycerophospholipid, phosphatidylinositol, and caffeine metabolism (Figure 4D,E). Heatmaps displayed the levels of metabolites associated with glycerophospholipid and phosphatidylinositol metabolism in each group (Figure 4F). Notably, compared to the Model group, the levels of two metabolites, 1‐hexadecyl‐sn‐glycero 3‐phosphate and 1‐stearoyl‐2‐linoleoyl‐sn‐glycerol, were significantly elevated in both the Control and POP groups (Figure 4G–I). These findings suggest that glycerolipid and inositol phosphate metabolisms may represent key metabolic pathways through which POP ameliorates aging, with 1‐hexadecyl‐sn‐glycero 3‐phosphate and 1‐stearoyl‐2‐linoleoyl‐sn‐glycerol potentially serving as key mediators in this process.

**FIGURE 4:**
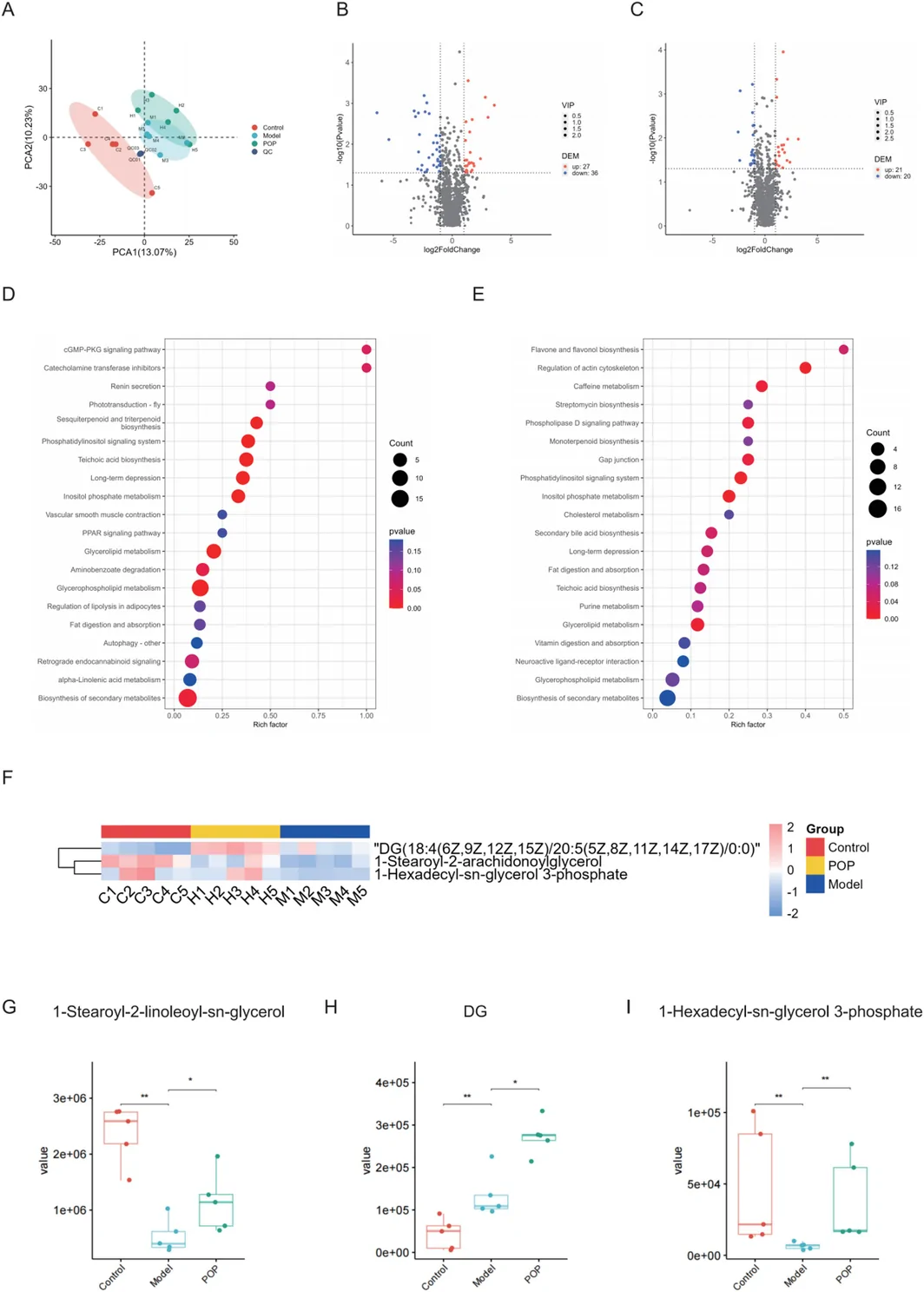
POP mediates alterations in serum metabolites in D‐galactose‐induced aging mice. (A) Principal component analysis (PCA) of all samples. (B) Volcano plot of differentially expressed metabolites between mice in the Control and Model groups. (C) Volcano plot of differentially expressed metabolites between mice in the POP and Model groups. (D) KEGG enrichment analysis of differentially expressed metabolites between mice in the Control and Model groups. (E) KEGG enrichment analysis of differentially expressed metabolites between mice in the POP and Model groups. (F) Heatmap of differentially expressed metabolites associated with glycerolipid metabolism and inositol phosphate metabolism. (G–I) Three differentially expressed metabolites related to glycerolipid and inositol phosphate metabolism. Data are presented as mean ± SD. *n* = 5. *p < 0.05, **p < 0.01.

### 3.4 POP Effects on Gene Expression in the Brains of D‐Gal‐Induced Aging Mice

Building on the observed alterations in serum metabolites, we sought to identify potentially affected genes and performed RNA sequencing to analyze the differences in gene expression profiles among the groups. PCA revealed a clear separation between mice in the Control and Model groups, whereas mice in the POP group exhibited a gene expression pattern that partially overlapped both groups (Figure 5A). Using the screening thresholds of |FoldChange| ≥ 2 and *p* ≤ 0.05, we identified 188 upregulated and 64 downregulated genes between mice in the Control and Model groups, and 118 upregulated and 55 downregulated genes between the mice in the POP and Model groups (Figure 5B,C). A Venn diagram shows 24 differentially expressed genes (DEGs) common to all three groups (Figure 5D). A heatmap further illustrated the expression levels of these DEGs across the groups, indicating that POP significantly altered the gene expression profile of D‐galactose‐induced aging mice (Figure 5E). Among the identified differentially expressed genes, *Chrdl2*, *Mybpc3*, and *Ninj2* were selected for further examination based on their RNA‐seq expression patterns. RNA‐seq showed higher expression of *Chrdl2* and *Mybpc3* and lower expression of *Ninj2* in the POP group than in the Model group (Figure 5F–H). RT‐qPCR analysis showed expression changes in *Mybpc3* and *Ninj2* that were consistent with the RNA‐seq results, whereas *Chrdl2* expression did not differ significantly among the three groups by RT‐qPCR (Figure 5I–K). Among the genes supported by both analyses, *Ninj2* showed the most pronounced expression change following POP treatment. These findings indicate that POP treatment was associated with altered hippocampal expression of *Mybpc3* and *Ninj2* in D‐galactose‐treated mice. The RNA‐seq finding for *Chrdl2* requires further validation.

**FIGURE 5:**
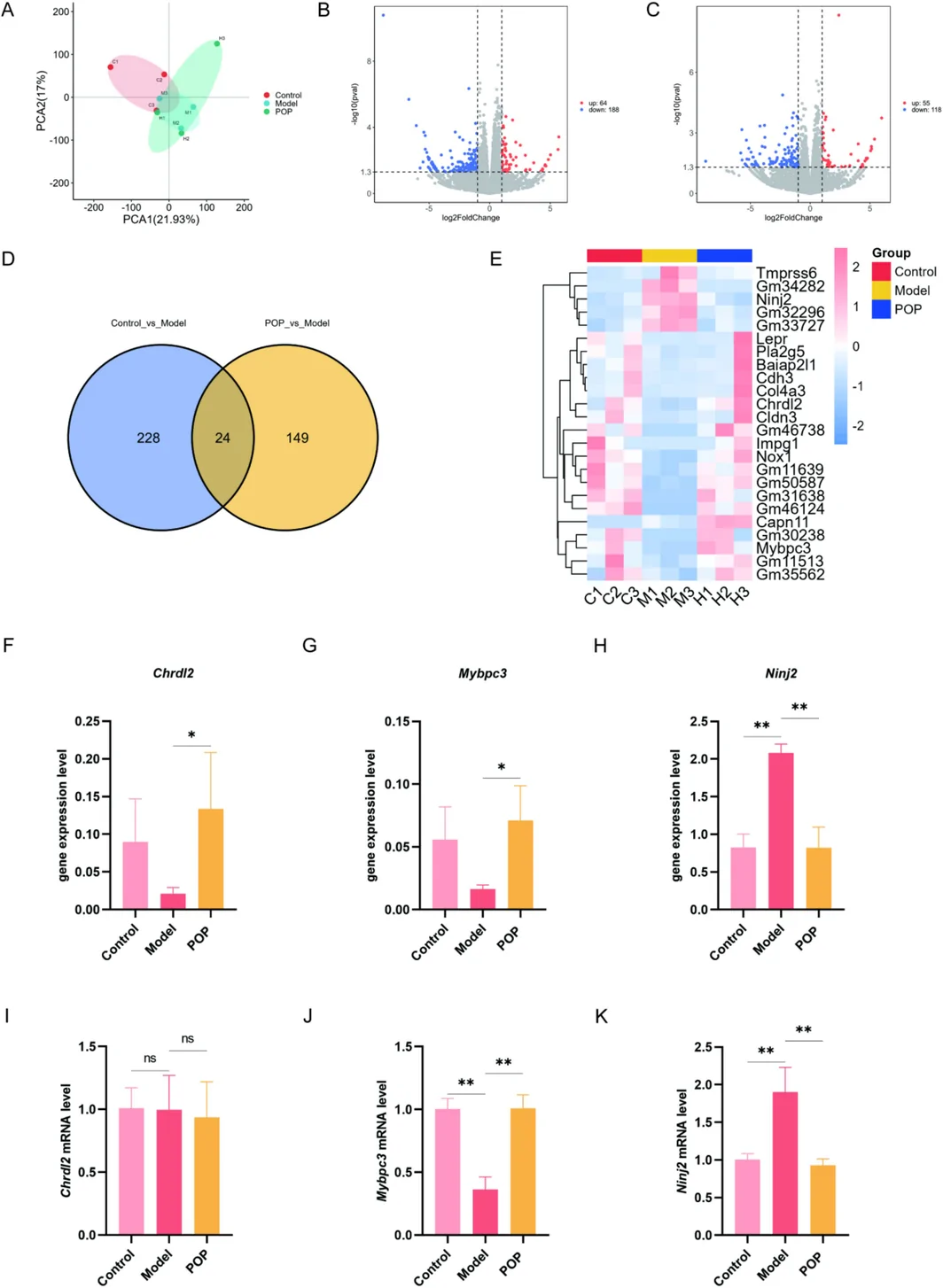
Effects of POP on gene expression in the brain of d‐galactose‐induced aging mice. (A) Principal component analysis (PCA) of all samples. (B) Volcano plot of differentially expressed genes (DEGs), Control vs. Model group. (C) Volcano plot of DEGs, POP versus Model group. (D) Venn diagram. (E) Heatmap of differentially expressed genes. (F–H) Expression levels of three significantly differentially expressed genes. (I–K) RT‐qPCR analysis of the selected genes. Data are presented as mean ± SD. *n* = 3. *p < 0.05, **p < 0.01.

### 3.5 POP Effects on Intestinal Gene Expression in D‐Gal‐Induced Aging Mice

To investigate the role of the brain‐gut axis in aging, we performed RNA sequencing to examine the differences in intestinal gene expression among different groups of mice. PCA revealed that, compared with mice in the Model group, those in the Control and POP groups exhibited distinct gene expression profiles (Figure 6A). Using the screening thresholds of |FoldChange| ≥ 2 and *p* ≤ 0.05, we identified 921 upregulated and 397 downregulated genes between the Control and Model groups, as well as 1647 upregulated and 895 downregulated genes between the POP and Model groups (Figure 6B,C). A Venn diagram showed 981 common DEGs among the three groups (Figure 6D). Further analysis revealed that these DEGs were significantly enriched in the NF‐κB signaling pathway, which is closely associated with aging (Figure 6E). Gene Set Enrichment Analysis (GSEA) further indicated that the NF‐κB signaling pathway was significantly activated in D‐galactose‐induced aging mice but markedly suppressed following POP treatment (Figure 6F). A heatmap displayed the expression levels of NF‐κB pathway‐related DEGs in different groups, demonstrating that POP significantly reversed the alterations induced by D‐galactose in aging mice (Figure 6G). Among the NF‐κB‐related genes identified by colonic RNA‐seq, Btk, Card11, Lat, and Tnfrsf13c were examined by RT‐qPCR. The RT‐qPCR results supported the RNA‐seq expression patterns of Btk, Lat, and Tnfrsf13c, whereas the pattern of Card11 was not confirmed (Figure 6I–L). Subsequent interpretation therefore focused on Btk, Lat, and Tnfrsf13c.

**FIGURE 6:**
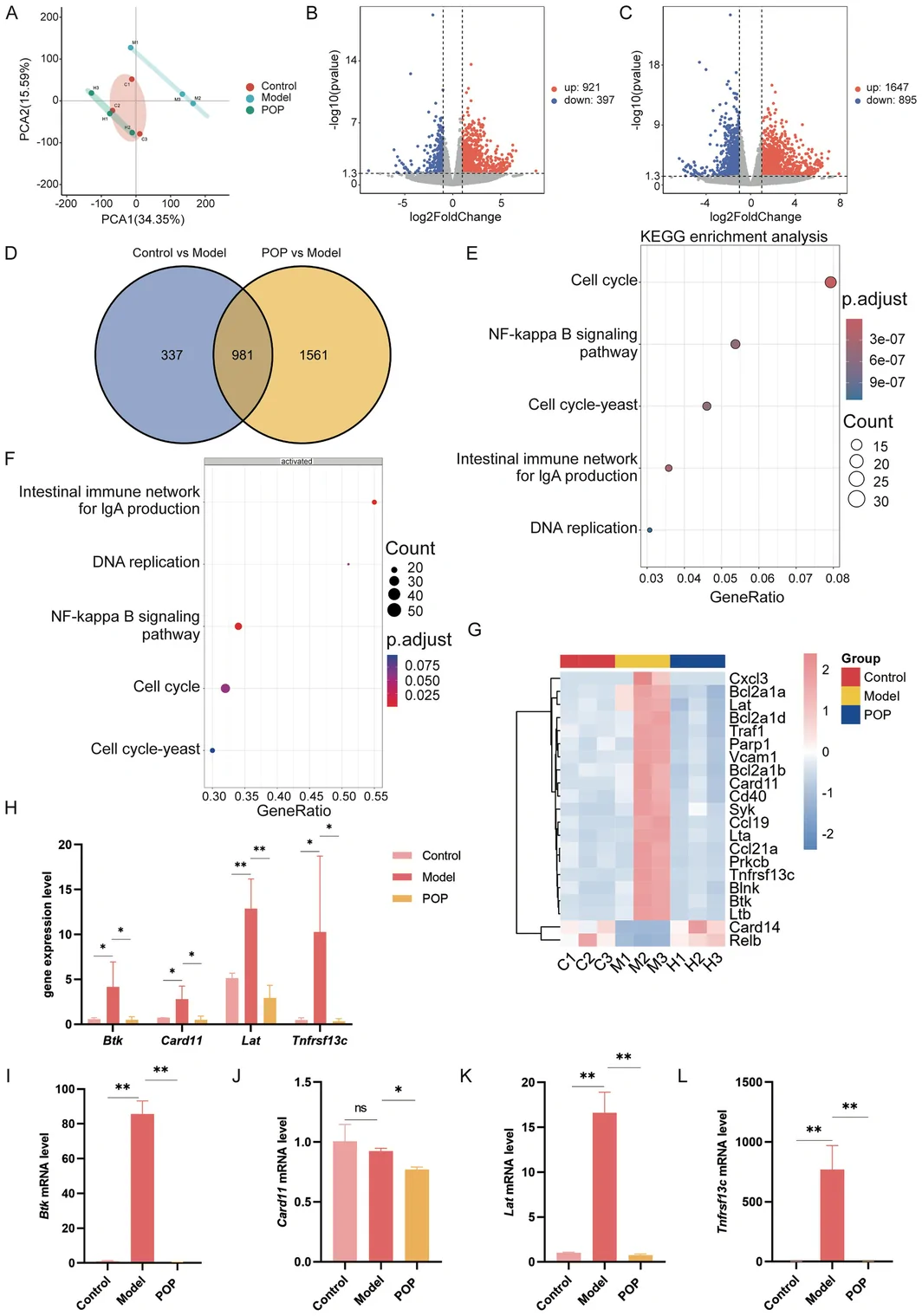
Effect of POP on intestinal gene expression in D‐galactose‐induced aging mice. (A) PCA analysis of all samples. (B) Volcano plot of differentially expressed genes (DEGs) in Control vs. Model group. (C) Volcano plot of DEGs in POP versus Model group. (D) Venn diagram. (E) KEGG enrichment analysis of DEGs. (F) GSEA of DEGs. (G) Heatmap of DEGs associated with the NF‐κB signaling pathway. (H) Four screened genes with significant differences. (I–L) RT‐qPCR analysis of the four screened genes with significant differences. Data are presented as mean ± SD. *n* = 3. *p < 0.05, **p < 0.01.

### 3.6 Comprehensive Analysis of Gut Microbiota, Metabolites, and Differentially Expressed Genes

To further explore the associations among differential gut microbiota, metabolites, and DEGs, we performed correlation analyses. At the genus level, correlation between serum metabolites involved in glycerolipid and inositol phosphate metabolism and differentially abundant gut microbiota (Figure 7A) revealed that the serum concentration of 1‐hexadecyl‐sn‐glycero 3‐phosphate, a metabolite associated with glycerophospholipid metabolism, was inversely correlated with the abundance of *Adlercreutzia*_404257, *Allobaculum*, and *Duncaniella*. Similarly, the concentration level of 1‐stearoyl‐2‐linoleoyl‐sn‐glycerol, a metabolite involved in both glycerolipid and inositol phosphate metabolism, was also negatively correlated with the abundance of these bacterial genera.

**FIGURE 7:**
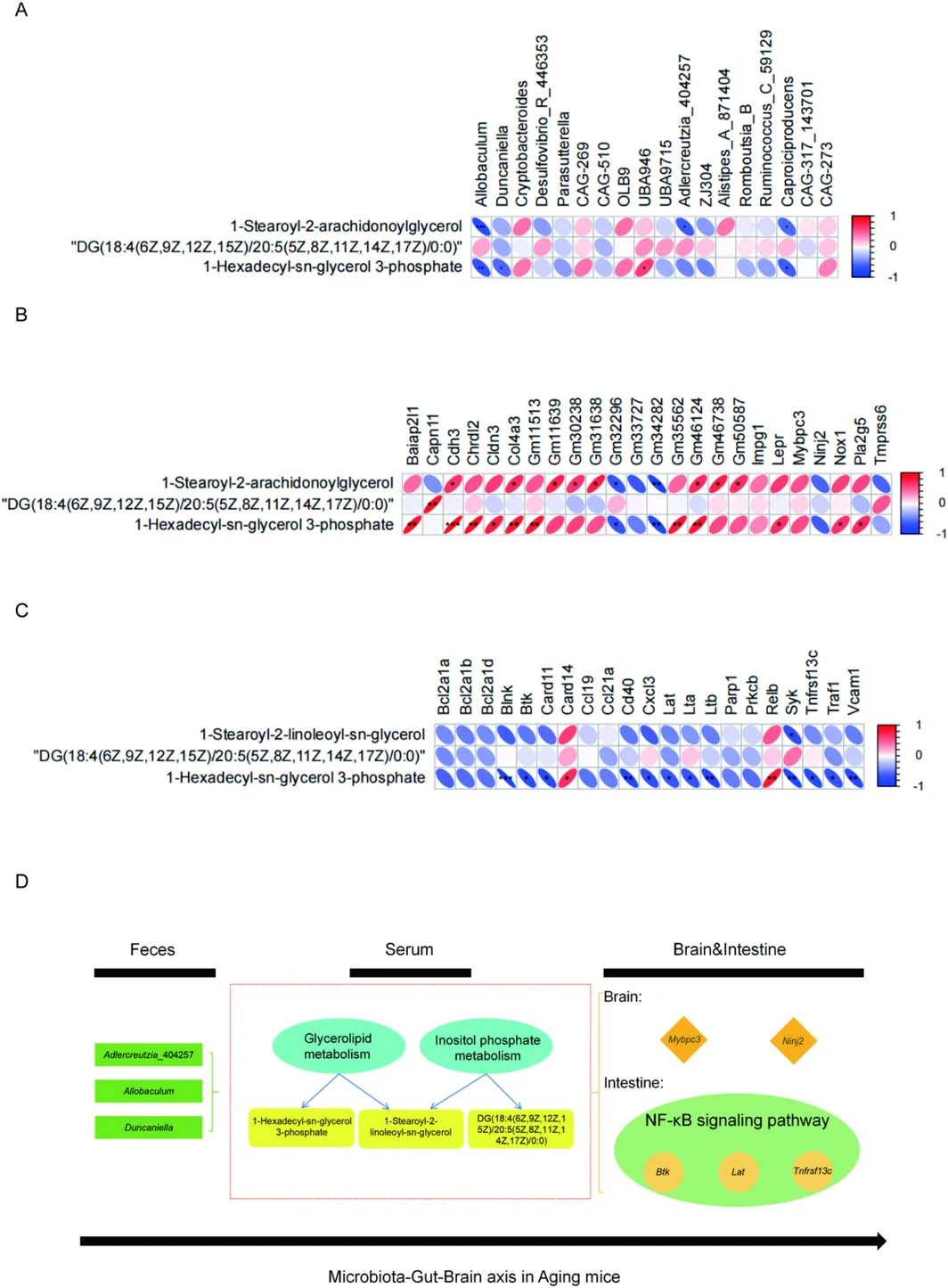
Integrated analysis of gut microbiota, metabolites, and differentially expressed genes. (A) Correlation elliptical heatmap between gut microbiota and serum metabolites. (B) Correlation elliptical heatmap between differentially expressed genes in the brain and serum metabolites. (C) Correlation elliptical heatmap between differentially expressed genes in the gut and serum metabolites; (D) Correlation network among gut microbiota, metabolites, and differentially expressed genes.

Furthermore, we examined the correlations between DEGs in the brain and gut and serum metabolites related to glycerolipid and inositol phosphate metabolism (Figure 7B). The results demonstrated that the expression levels of *Chrdl2* and *Mybpc3* were positively correlated with both the levels of 1‐hexadecyl‐sn‐glycero 3‐phosphate and 1‐stearoyl‐2‐linoleoyl‐sn‐glycerol, whereas *Ninj2* exhibited the opposite trend.

Regarding intestinal genes, key genes closely associated with the activation of the NF‐κB signaling pathway, such as *Btk*, *Lat*, and *Tnfrsf13c*, were also negatively correlated with the levels of 1‐hexadecyl‐sn‐glycero 3‐phosphate and 1‐stearoyl‐2‐linoleoyl‐sn‐glycerol (Figure 7C). Figure 7D presents a schematic summary of the findings across the fecal microbiota, serum metabolite, hippocampal transcriptomic, and colonic transcriptomic datasets. It highlights the selected bacterial taxa, metabolites, and genes described in Figure 7A–C, including *Mybpc3* and *Ninj2* in the hippocampus and *Btk*, *Lat*, and *Tnfrsf13c* in the colon. The schematic summarizes concurrent changes and cross‐omics correlations observed in this study rather than a confirmed sequence of biological regulation.

Collectively, these findings suggest that POP may mitigate the aberrant increase in gut microbiota, such as *Adlercreutzia*_404257, *Allobaculum*, and *Duncaniella*, in aged mice, and regulate systemic homeostasis of glycerolipid and inositol phosphate metabolism.

## 4 Discussion

Life is a continuous process of aging, characterized by a gradual decline in physiological functions and organ systems over time (Mazhar et al. 2021). Aging is recognized as a major risk factor for neurodegenerative diseases such as AD and Parkinson's disease, with the likelihood of developing these conditions increasing significantly with age (Hou et al. 2019; Jiang et al. 2025). Natural polysaccharide extracts have significant anti‐aging effects, and their mechanisms of action may be related to their antioxidant and immunomodulatory activities (Huang et al. 2025; Ma et al. 2021). However, systematic research on the pathways through which polysaccharides exert these effects remains limited. Integrated multi‐omics association studies, which combine multi‐level biological molecule data from genomics, transcriptomics, proteomics, metabolomics, and epigenomics, have been widely used to explore the pathogenesis of complex diseases and have demonstrated capabilities surpassing those of single‐omics analyses (Behrens et al. 2026). Therefore, in this study, we employed an integrated multi‐omics approach to investigate the protective effects of POP on aging mice from three perspectives: gut microbiota, serum metabolites, and the transcriptome. Our findings indicate that POP reduces the abnormal increase in genera such as *Adlercreutzia*_404257, *Allobaculum*, and *Duncaniella* in the intestines of aging mice, regulates glycerolipid metabolism and inositol phosphate metabolism homeostasis in vivo, and its mechanism of action may be related to modulating the expression of genes associated with oxidative stress (such as *Ninj2*, *Btk*, *Lat*, and *Tnfrsf13c*) in the brain and gut.

Notably, POP treatment significantly ameliorated neuronal damage in the hippocampal CA1 region, a key area for learning and memory. The restoration of Nissl bodies and neuronal integrity suggests that POP enhances cell survival and structural maintenance. Concurrently, POP significantly alleviated oxidative stress, as evidenced by the reduced MDA levels and increased GSH and SOD activities. These findings are consistent with our previous research, further supporting the conclusion that the antioxidant properties of POP are a key mechanism underlying its neuroprotective effects.

Furthermore, our findings indicate that POP modulates the gut microbiota, as evidenced by changes in alpha and beta diversity, as well as alterations in specific genera, such as *Adlercreutzia*_404257, *Allobaculum*, and *Duncaniella*. In a study investigating the relationship between gut microbiota and AD, *Adlercreutzia* was reported to be closely associated with AD (Cammann et al. 2023). Similarly, a study on the impact of a high‐fat diet on aging demonstrated that a high‐fat diet disrupts the gut microbial composition, leading to a significant increase in *Allobaculum* bacteria in the intestines of aging mice. This increase was associated with the development of systemic non‐resolving inflammation in acute heart failure, accompanied by the disruption of immune cell distribution (Kain et al. 2019). Although no studies have directly linked *Duncaniella* to aging, research has found that genetically identical mice differ in susceptibility to inflammatory bowel disease, and this variation is associated with specific gut microbes, including *Duncaniella*; these findings suggest that *Duncaniella* may contribute to the promotion of inflammation (Forster et al. 2022). In our study, the relative abundances of *Adlercreutzia*_404257, *Allobaculum*, and *Duncaniella* were abnormally increased in aging mice, whereas POP treatment restored their levels close to normal. Therefore, POP treatment was associated with reduced abundance of these genera; however, whether this regulation causally contributes to its anti‐aging effects requires further validation. Collectively, while the reduction of *Adlercreutzia*_404257, *Allobaculum*, and *Duncaniella* by POP correlates with improved aging phenotypes, the precise contribution of each genus requires future mechanistic validation.

To further investigate the impact of gut microbial changes on the host metabolite levels, our untargeted metabolomics analysis revealed that POP primarily ameliorated aging by modulating glycerolipid and inositol phosphate metabolism. Based on this finding, we identified two key metabolites involved in these pathways: 1‐hexadecyl‐sn‐glycerol 3‐phosphate and 1‐stearoyl‐2‐linoleoyl‐sn‐glycerol. 1‐hexadecyl‐sn‐glycerol 3‐phosphate is a precursor or analog of lysophosphatidic acid (LPA), an important lipid signaling molecule that regulates cell proliferation, differentiation, and survival. Maintaining LPA homeostasis is essential for the stability of cellular signaling pathways. 1‐stearoyl‐2‐linoleoyl‐sn‐glycerol is a diacylglycerol (DAG), a key lipid signaling molecule that primarily activates the protein kinase C pathway and regulates cell growth, secretion, and inflammatory responses. Moreover, DAG serves as an important intermediate in the synthesis of complex phospholipids, such as phosphatidylinositol and glycerophospholipids. In our study, the levels of these two metabolites were lower than normal in the serum of aging mice and were restored to normal levels after POP treatment. This finding suggests that cells regain the capacity to synthesize membrane phospholipids with normal structure and healthy fatty acid ratios, enhancing resistance to oxidative stress and reducing lipid peroxidation.

Transcriptomic analysis of the hippocampus identified POP‐associated changes in *Chrdl2*, *Mybpc3*, and *Ninj2*. However, the RNA‐seq finding for *Chrdl2* was not confirmed by RT‐qPCR, which showed no significant difference among the groups. CHRDL2 encodes chordin‐like 2, an antagonist of bone morphogenetic protein (BMP) signaling (Clarkson and Lewis 2025), but its role in hippocampal aging or neuronal injury remains unclear. We therefore regard *Chrdl2* as an exploratory RNA‐seq finding. The changes in *Mybpc3* and *Ninj2* were supported by RT‐qPCR. *Mybpc3* has been studied predominantly in cardiac muscle, where its alteration has been associated with mitochondrial dysfunction and increased reactive oxygen species (Cao et al. 2025; Lynch 4th et al. 2015). Its function in the hippocampus remains unknown, so the cardiac findings cannot establish a similar role in our model. NINJ2 belongs to the nerve injury‐induced protein family (Zhang et al. 2024). Previous experimental work has implicated NINJ2 in neuroinflammation and oxidative stress through TLR4/NF‐κB‐related signaling (Liu et al. 2025). In our study, POP treatment was associated with lower hippocampal *Ninj2* expression and improved cerebral oxidative stress markers, making *Ninj2* a candidate for future functional investigation. In the cross‐omics analysis, the serum metabolites shown in Figure 6B were positively correlated with hippocampal *Mybpc3* expression and negatively correlated with *Ninj2* expression. An association with *Chrdl2* was also observed using the RNA‐seq data, but this finding should be interpreted cautiously because the group difference in *Chrdl2* expression was not confirmed by RT‐qPCR. These correlations do not show that the metabolites regulate hippocampal gene expression. Likewise, hippocampal *Ninj2* expression and the colonic NF‐κB‐related transcriptional changes were observed in different tissues; their mechanistic relationship was not tested in this study.

However, we observed similar mechanisms in the gut. KEGG enrichment analysis of DEGs revealed significant enrichment in the NF‐κB signaling pathway. Notably, this pathway was significantly activated in aging mice and was associated with markedly increased expression of *Btk*, *Lat*, and *Tnfrsf13c*. BTK is a key tyrosine kinase downstream of B‐cell receptor and Toll‐like receptor signaling that activates NF‐κB and contributes to intestinal inflammation and mucosal immune regulation (Mao et al. 2020). BTK also supports intestinal IgA responses, and BTK‐deficient mice exhibit reduced IgA coating of commensal bacteria and altered colonization of specific taxa such as *Parabacteroides distasonis* (Bonami et al. 2022), indicating a functional link between *Btk* and gut microbiota. LAT is a key adaptor protein in T‐cell receptor signaling that participates in T‐cell activation and NF‐κB signaling (Balagopalan et al. 2010). Downregulation of *Lat* in natural intestinal intraepithelial T lymphocytes is considered a tolerance checkpoint that prevents excessive activation against self‐antigens and commensal microbiota. However, no direct evidence currently links *Lat* expression or function to gut microbiota composition. TNFRSF13C (BAFF‐R) mediates B‐cell survival and NF‐κB activation and participates in the intestinal IgA immune network. Probiotic *Bacillus amyloliquefaciens* can downregulate ileal *Tnfrsf13c* expression (Hong et al. 2021). These observations suggest that *Tnfrsf13c* may link gut microbiota to intestinal NF‐κB signaling and that its upregulation in aging mice may be associated with gut dysbiosis. These findings provide a possible immunological context for the concurrent changes in colonic gene expression and gut bacterial composition observed in our study. Oxidative stress can contribute to NF‐κB activation, and NF‐κB regulates genes involved in inflammatory and cellular stress responses (Ya and Bayraktutan 2024). Sustained oxidative stress and inflammation during aging may therefore be accompanied by altered NF‐κB‐related transcription (Li et al. 2025). Following POP treatment, the colonic expression of *Btk*, *Lat*, and *Tnfrsf13c* decreased, together with an attenuated NF‐κB‐related transcriptional signature. These changes occurred alongside alterations in the relative abundance of selected gut bacterial taxa. Whether mucosal immune signaling contributes to those bacterial changes, or the bacterial changes influence colonic immune signaling, remains to be determined.

Finally, the integrated analysis identified associations across the gut bacterial, serum metabolomic, and tissue transcriptomic datasets. POP treatment was accompanied by changes in the relative abundances of selected bacterial taxa, including Adlercreutzia_404257, Allobaculum, and Duncaniella, and in circulating lipid metabolites. The selected metabolites were correlated with hippocampal expression of *Mybpc3* and *Ninj2* and with colonic expression of NF‐κB‐related genes, including *Btk*, *Lat*, and *Tnfrsf13c*. These observations provide a basis for examining possible links among mucosal immune responses, circulating metabolism, and hippocampal responses. They do not demonstrate that the bacterial changes produced the metabolite changes or that the metabolites regulated gene expression in either tissue.

One possible interpretation is that changes in gut bacterial composition and mucosal immune activity occur alongside changes in circulating metabolites, which may be relevant to systemic inflammatory and oxidative stress responses. The measured correlations are consistent with this possibility, but the sequence and direction of these events remain unknown. In particular, the present study did not test whether altered gut microbiota are necessary for POP‐associated changes in serum metabolites, colonic gene expression, hippocampal gene expression, or oxidative stress markers. Microbiota depletion or fecal microbiota transplantation, followed by targeted manipulation of candidate genes or pathways, will be needed to assess the causal contributions of these components. The D‐galactose‐induced model captures selected oxidative stress‐related features of aging but does not reproduce the gradual progression of physiological aging. In particular, the eight‐week model may not reflect the long‐term succession of gut microbial communities or the chronic inflammatory changes that develop during natural aging. Our findings should therefore be interpreted in the context of D‐galactose‐treated mice, and their relevance to physiological aging requires validation in naturally aged animals.

Biologically, this chain of associations can be interpreted as follows. POP‐associated remodeling of the gut bacterial community may alter the production or absorption of circulating lipid metabolites, particularly glycerophospholipid‐ and inositol phosphate‐related species. These metabolites may in turn influence host gene expression in the intestine and brain, either directly through lipid signaling or indirectly by modulating oxidative stress and inflammatory pathways. In the intestine, the levels of these metabolites were negatively correlated with NF‐κB‐related genes (*Btk*, *Lat*, and *Tnfrsf13c*), suggesting that microbiota‐modulated lipid signals may contribute to the suppression of intestinal NF‐κB activation. In the brain, the same metabolites were positively correlated with *Chrdl2* and *Mybpc3* and negatively correlated with *Ninj2*, suggesting that systemic lipid signaling may be associated with cerebral oxidative stress‐related gene expression. However, these correlations do not establish directionality or causality. Future studies involving microbiota depletion, fecal microbiota transplantation, or targeted functional validation are needed to determine whether changes in gut microbiota causally drive the observed metabolite and gene expression changes. The molecular weight distribution of the POP preparation used in this study was not determined. Further characterization of its molecular weight distribution will be important for comparing POP preparations across studies and investigating potential structure–activity relationships.

In summary, this study provides multi‐omics evidence that POP treatment is associated with amelioration of D‐galactose‐induced aging and with changes in interconnected pathways involving oxidative stress, gut microbiota, serum metabolism, and gene expression. The multi‐target and system‐level actions of these highlight the potential of POP as a natural therapeutic agent against aging and associated cognitive impairments. Future research should aim to validate these mechanisms in higher organisms and elucidate the precise molecular interactions between POP and its key targets.

## 5 Conclusions

In D‐galactose‐treated mice, POP treatment was associated with improved hippocampal histology and oxidative stress markers, together with changes in gut bacterial composition, serum metabolites, and hippocampal and colonic gene expression. Cross‐omics analysis identified correlations among selected bacterial taxa, circulating lipid metabolites, hippocampal *Mybpc3* and *Ninj2*, and colonic NF‐κB‐related genes. These findings identify candidate gut–serum–tissue associations for further investigation. Whether the microbiota, metabolites, or tissue gene‐expression changes mediate the observed effects of POP remains to be determined through functional studies.

## Author Contributions

**Wenjian Li:** writing – original draft. **Yan Huang:** methodology, funding acquisition. **Wenfang Zhang:** investigation. **Shunxiang Li:** writing – review and editing. **Lisong Li:** visualization, supervision.

## Funding

The research supported by National Natural Science Foundation of Hunan Province (2025JJ80185, 2024JJ8172), Research Project of the Administration of Traditional Chinese Medicine of Hunan Province (A2023006), National Natural Science Foundation of China (82204924).

## Ethics Statement

The study protocol was approved by the Institutional Animal Care and Use Committee (IACUC) of Hunan University of Chinese Medicine (ethical batch number: SLBH‐202410300002), in line with the welfare and ethical principles of laboratory animals.

## Conflicts of Interest

The authors declare no conflicts of interest.

## Data Availability

The data that support the findings of this study are available from the corresponding author upon reasonable request.

## References

[fsn372500-bib-0001] Balagopalan, L. , N. P. Coussens , E. Sherman , L. E. Samelson , and C. L. Sommers . 2010. “The LAT Story: A Tale of Cooperativity, Coordination, and Choreography.” Cold Spring Harbor Perspectives in Biology 2, no. 8: a005512. 10.1101/cshperspect.a005512. PMC2908767 20610546

[fsn372500-bib-0002] Behrens, L. M. P. , G. D. S. Fernandes , G. F. Gonçalves , et al. 2026. “Limitations and Opportunities in Multi‐Omics Integration for Neurodevelopmental, Neurodegenerative and Psychiatric Disorders: A Systematic Review.” Neuroscience 599: 76–93. 10.1016/j.neuroscience.2026.01.019. 41638346

[fsn372500-bib-0003] Bonami, R. H. , C. E. Thurman , S. Verma , et al. 2022. “Bruton's Tyrosine Kinase Supports Gut Mucosal Immunity and Commensal Microbiome Recognition in Autoimmune Arthritis.” Frontiers in Immunology 13: 748284. 10.3389/fimmu.2022.748284. PMC9002138 35422819

[fsn372500-bib-0004] Cammann, D. , Y. Lu , and M. J. Cummings . 2023. “Genetic Correlations Between Alzheimer's Disease and Gut Microbiome Genera.” Scientific Reports 13: 5258. 10.1038/s41598-023-31730-5. PMC10066300 37002253

[fsn372500-bib-0005] Cao, J. , Y. Zhai , K. Li , et al. 2025. “ROS Accelerates the Progression of Hypertrophic Cardiomyopathy.” Genes & Diseases 13, no. 1: 101741. 10.1016/j.gendis.2025.101741. PMC12555768 41158753

[fsn372500-bib-0006] Clarkson, E. , and A. Lewis . 2025. “BMP Antagonist CHRDL2 Enhances the Cancer Stem‐Cell Phenotype and Increases Chemotherapy Resistance in Colorectal Cancer.” Molecular Oncology 19, no. 11: 3135–3155. 10.1002/1878-0261.70064. PMC12591322 40434957

[fsn372500-bib-0007] Dong, R. , Q. Wu , J. Kan , et al. 2026. “Insights Into the Therapeutic Strategies for Aging and Aging‐Associated Diseases.” Signal Transduction and Targeted Therapy 11, no. 1: 202. 10.1038/s41392-026-02662-z. PMC13226726 42225652

[fsn372500-bib-0008] Forster, S. C. , S. Clare , and B. S. Beresford‐Jones . 2022. “Identification of Gut Microbial Species Linked With Disease Variability in a Widely Used Mouse Model of Colitis.” Nature Microbiology 7: 590–599. 10.1038/s41564-022-01094-z. PMC8975739 35365791

[fsn372500-bib-0009] Hong, Y. , Y. Cheng , L. Guan , et al. 2021. “ *Bacillus amyloliquefaciens* TL Downregulates the Ileal Expression of Genes Involved in Immune Responses in Broiler Chickens to Improve Growth Performance.” Microorganisms 9, no. 2: 382. 10.3390/microorganisms9020382. PMC7918048 33668643

[fsn372500-bib-0010] Hou, Y. , X. Dan , M. Babbar , et al. 2019. “Ageing as a Risk Factor for Neurodegenerative Disease.” Nature Reviews Neurology 15: 565–581. 10.1038/s41582-019-0244-7. 31501588

[fsn372500-bib-0011] Huang, R. , R. Li , M. Qin , et al. 2025. “A Review on Anti‐Aging Actions of Plant Polysaccharides.” Future Foods 12: 100684. 10.1016/j.fufo.2025.100684.

[fsn372500-bib-0012] Jiang, Q. , J. Liu , S. Huang , et al. 2025. “Antiageing Strategy for Neurodegenerative Diseases: From Mechanisms to Clinical Advances.” Signal Transduction and Targeted Therapy 10, no. 1: 76. 10.1038/s41392-025-02145-7. PMC11891338 40059211

[fsn372500-bib-0013] Kain, V. , W. Van Der Pol , and N. Mariappan . 2019. “Obesogenic Diet in Aging Mice Disrupts Gut Microbe Composition and Alters Neutrophil:Lymphocyte Ratio, Leading to Inflamed Milieu in Acute Heart Failure.” FASEB Journal 33: 6456–6469. 10.1096/fj.201802477R. PMC6463911 30768364

[fsn372500-bib-0014] Kroemer, G. , A. B. Maier , and A. M. Cuervo . 2025. “From Geroscience to Precision Geromedicine: Understanding and Managing Aging.” Cell 188: 2043–2062. 10.1016/j.cell.2025.03.011. PMC12037106 40250404

[fsn372500-bib-0015] Li, Z. , T. Wang , and S. Du . 2025. “Tgm2‐Catalyzed Covalent Cross‐Linking of IκBα Drives NF‐κB Nuclear Translocation to Promote SASP in Senescent Microglia.” Aging Cell 24: e14463. 10.1111/acel.14463. PMC12073898 39749582

[fsn372500-bib-0016] Liu, S. , Y. He , H. Chen , et al. 2025. “SLAMF8 and NINJ2 Promote Neuroinflammation and Oxidative Stress Through TLR4 NF‐Kappa B Pathway in Alzheimer's Disease.” Scientific Reports 15, no. 1: 17501. 10.1038/s41598-025-02097-6. PMC12092773 40394132

[fsn372500-bib-0017] Lynch, T. L., 4th , M. Sivaguru , M. Velayutham , et al. 2015. “Oxidative Stress in Dilated Cardiomyopathy Caused by MYBPC3 Mutation.” Oxidative Medicine and Cellular Longevity 2015: 424751. 10.1155/2015/424751. PMC4609873 26508994

[fsn372500-bib-0018] Ma, W. , S. Wei , and W. Peng . 2021. “Antioxidant Effect of Polygonatum Sibiricum Polysaccharides in D‐Galactose‐Induced Heart Aging Mice.” BioMed Research International 2021: 6688855. 10.1155/2021/6688855. PMC8024086 33860051

[fsn372500-bib-0019] Mao, L. , A. Kitani , E. Hiejima , et al. 2020. “Bruton Tyrosine Kinase Deficiency Augments NLRP3 Inflammasome Activation and Causes IL‐1β‐Mediated Colitis.” Journal of Clinical Investigation 130: 1793–1807. 10.1172/JCI128322. PMC7108929 31895698

[fsn372500-bib-0020] Mazhar, M. , A. U. Din , and H. Ali . 2021. “Implication of Ferroptosis in Aging.” Cell Death Discovery 7: 149. 10.1038/s41420-021-00553-6. PMC8257567 34226536

[fsn372500-bib-0021] Peng, Z. , H. Li , S. Peng , C. He , J. Yi , and L. Xiao . 2020. “The Effect of *Polygonatum odoratum* Polysaccharide on Cognitive Impairment of D‐Galactose‐Induced Aging Mice.” Chinese Archives of Traditional Chinese Medicine 38, no. 11: 254–258. 10.13193/j.issn.1673-7717.2020.11.061.

[fsn372500-bib-0022] Ren, X. , J. Ma , Y. Zhao , et al. 2026. “Microbiome Remodeling During Aging: Integrative Multi‐Omics and Spatiotemporal Perspectives on Immune and Metabolic Regulation.” Ageing Research Reviews 121: 103269. 10.1016/j.arr.2026.103269. 42480622

[fsn372500-bib-0023] Shu, Q. , B. Liao , Z. Zhu , et al. 2026. “Botany, Traditional Uses, Phytochemistry, Pharmacology, Toxicology, and Applications of the Genus Polygonatum: A Comprehensive Update Review.” Journal of Ethnopharmacology 368: 121773. 10.1016/j.jep.2026.121773. 42092474

[fsn372500-bib-0024] Wang, Z. , S. Ge , and T. Liao . 2025. “Integrative Single‐Cell Metabolomics and Phenotypic Profiling Reveals Metabolic Heterogeneity of Cellular Oxidation and Senescence.” Nature Communications 16, no. 1: 2740. 10.1038/s41467-025-57992-3. PMC11926267 40113759

[fsn372500-bib-0025] Xiao, L. , H. Wen , and S. Peng . 2024. “Polygonatum Polysaccharide Ameliorates D‐Galactose‐Induced Cognitive Dysfunction in Aging Rats by Inhibiting Ferroptosis Through Activation of Nrf2.” Neuroscience Letters 836: 137873. 10.1016/j.neulet.2024.137873. 38871020

[fsn372500-bib-0026] Ya, J. , and U. Bayraktutan . 2024. “Senolytics and Senomorphics Targeting p38MAPK/NF‐κB Pathway Protect Endothelial Cells From Oxidative Stress‐Mediated Premature Senescence.” Cells 13: 1292. 10.3390/cells13151292. PMC11311971 39120322

[fsn372500-bib-0027] Yang, H. , T. Wang , and C. Qian . 2025. “Gut Microbial‐Derived Phenylacetylglutamine Accelerates Host Cellular Senescence.” Nature Aging 5: 401–418. 10.1038/s43587-024-00795-w. 39794469

[fsn372500-bib-0028] Yang, S. , Z. Wei , and J. Luo . 2024. “Integrated Bioinformatics and Multiomics Reveal Liupao Tea Extract Alleviating NAFLD via Regulating Hepatic Lipid Metabolism and Gut Microbiota.” Phytomedicine 132: 155834. 10.1016/j.phymed.2024.155834. 38941818

[fsn372500-bib-0029] Zhang, J. , X. Kong , H. J. Yang , W. Zhang , M. Chen , and X. Chen . 2024. “Ninjurin 2 Modulates Tumorigenesis, Inflammation, and Metabolism via Pyroptosis.” American Journal of Pathology 194: 849–860. 10.1016/j.ajpath.2024.01.013. PMC11074982 38325550

[fsn372500-bib-0030] Zhao, X. , J. Xiu , and H. Yang . 2025. “Network Pharmacology and Bioinformatics Study of Six Medicinal Food Homologous Plants Against Colorectal Cancer.” International Journal of Molecular Sciences 26, no. 3: 930. 10.3390/ijms26030930. PMC11817456 39940699

